# Fenofibrate Mitigates Acute Lung Injury in a Rat Model of Feces-Induced Peritonitis

**DOI:** 10.3390/ijms27083556

**Published:** 2026-04-16

**Authors:** Ahmet Akbaş, Mehmet Fatih Dasiran, Hassen Daghmoura, Bakiye Akbaş, Hatice Aygun, Ahmet Serdaroglu, Yiğit Uyanikgil, Gülçin Ercan, Oytun Erbas

**Affiliations:** 1 Department of General Surgery, Faculty of Medicine, Karadeniz Technical University, 61080 Trabzon, Türkiye; 2 Department of General Surgery, Faculty of Medicine, Tokat Gaziosmanpasa University, 60250 Tokat, Türkiye; fatihdasiran@yahoo.com; 3 Department of General Surgery, Faculty of Medicine, Bezmialem Vakif University, 34093 Istanbul, Türkiye; hasgagh@hotmail.com; 4 Department of Obstetrics and Gynecology, Faculty of Medicine, Karadeniz Technical University, 61080 Trabzon, Türkiye; bakiyeakbas@ktu.edu.tr; 5 Neuroscience Laboratory, BAMER, Biruni University, 34010 Istanbul, Türkiye; hatice_5aygun@hotmail.com; 6 Department of Internal Medicine, Faculty of Medicine, Karadeniz Technical University, 61080 Trabzon, Türkiye; serdaroglu@ktu.edu.tr; 7 Department of Histology and Embryology, Faculty of Medicine, Ege University, 35100 Izmir, Türkiye; yigit.uyanikgil@ege.edu.tr; 8 Department of General Surgery, Sultan 2. Abdulhamid Han Educational and Research Hospital, Istanbul Provincial Directorate of Health, 34668 Istanbul, Türkiye; gulcin.ercan@sbu.edu.tr; 9 Faculty of Medicine, BAMER, Biruni University, 34010 Istanbul, Türkiye; oytunerbas2012@gmail.com

**Keywords:** fenofibrate, sepsis, fecal intraperitoneal injection (FIP), acute lung injury, HSP70, Nrf2, oxidative stress, inflammation, cytoprotection, rat model

## Abstract

This study aimed to investigate the protective effects of fenofibrate against sepsis-induced acute lung injury using a feces-induced peritonitis (FIP) rat model, with particular emphasis on the modulation of HSP70 and Nrf2 as key cellular defense mechanisms. The FIP model was employed to mimic colon-origin abdominal sepsis, frequently encountered in general surgery, including conditions such as colonic perforation and anastomotic leakage. Thirty male Wistar albino rats were randomly assigned to control, FIP, and FIP + fenofibrate groups. Sepsis was induced by intraperitoneal injection of a fecal-saline suspension. Fenofibrate (100 mg/kg) was administered intraperitoneally after the FIP procedure. After 24 h, lung tissues and blood samples were collected. Assessments included histopathology (H&E staining), thoracic CT imaging, arterial blood gas analysis, ELISA-based quantification of plasma cytokines (IL-6, IL-1β, TNF-α), MDA for oxidative stress, and lung tissue levels of HSP70 and Nrf2. Feces-induced peritonitis caused severe acute lung injury, evidenced by increased histopathological damage (*p* < 0.001), impaired gas exchange (PaO_2_ and PaCO_2_, *p* < 0.01), elevated inflammatory cytokines (IL-6, IL-1β, TNF-α; *p* < 0.001), increased oxidative stress (MDA, *p* < 0.001), and suppressed lung Nrf2 and HSP70 expression (*p* < 0.001). Fenofibrate significantly attenuated lung injury, improved gas exchange (*p* < 0.05), reduced inflammation (*p* < 0.01–*p* < 0.001), decreased MDA (*p* < 0.001), and increased Nrf2 (*p* < 0.001) and HSP70 (*p* < 0.01). Fenofibrate attenuates sepsis-induced acute lung injury by reducing inflammation and oxidative stress while preserving HSP-70 and Nrf2-mediated cytoprotective pathways. These findings are clinically relevant to general surgery, as septic lung injury commonly arises from colon-origin abdominal sepsis, including colonic perforation and anastomotic leakage.

## 1. Introduction

Sepsis remains a major global health problem and a leading cause of death and long-term morbidity [1]. Acute respiratory distress syndrome (ARDS) is a severe manifestation of sepsis-associated acute lung injury and continues to carry high mortality in critical care settings [2,3]. Reported ARDS rates among patients with sepsis vary substantially because they depend on illness severity, clinical context, and the case definition applied; incidence is typically lower in broad sepsis cohorts but increases in more severely ill subgroups such as septic shock [4,5]. ARDS is not limited to adults; pediatric acute respiratory distress syndrome (PARDS) is also commonly triggered by infection, and nonpulmonary sepsis represents a major indirect etiology in pediatric cohorts [6,7,8].

Clinically, ARDS most often follows pneumonia, aspiration, major trauma, or nonpulmonary sepsis, and peritoneal infection is recognized among extrapulmonary sepsis sources that can precipitate ARDS [3]. In the multinational LUNG SAFE cohort, extrapulmonary sepsis accounted for approximately 16% of identified ARDS risk factors [2]. From a surgical perspective, intra-abdominal sepsis is frequent and has been described as the second most common source of sepsis [9]; recent ICU data further suggest that ARDS may develop in a substantial proportion of patients with intra-abdominal sepsis [10].

According to the Berlin definition, ARDS develops within 1 week of a known clinical insult, and most cases are identified within 72 h after recognition of the underlying risk factor [11]. Because experimental models cannot fully reproduce the clinical complexity of human ARDS, consensus recommendations emphasize documenting experimental acute lung injury across complementary domains, including histologic injury, alveolar-capillary barrier alteration, inflammatory response, and physiologic dysfunction, using time windows appropriate for the modeled insult [12,13]. Consistent with this framework, we used a feces-induced peritonitis (FIP) model to generate indirect, abdominal polymicrobial sepsis and to evaluate early lung injury within 24 h. In rats, lung involvement in this model evolves rapidly and becomes prominent within the first day, enabling assessment of early ARDS-like endpoints in a clinically relevant sepsis context [14,15].

Despite different initiating insults, acute lung injury (ALI) and ARDS share convergent pathogenic mechanisms, including dysregulated innate immune activation, excessive cytokine and chemokine release, neutrophil recruitment, and oxidative and nitrosative stress. These processes injure the alveolar epithelium and pulmonary endothelium, increase alveolar-capillary permeability, and lead to pulmonary edema with impaired gas exchange [3,16,17]. In sepsis-associated ALI, the inflammatory cascade is amplified by elevated levels of pro-inflammatory cytokines such as TNF-α, IL-1β, and IL-6, accompanied by endothelial dysfunction and disruption of alveolar-capillary barrier integrity [18,19]. Within this inflammatory and oxidant-rich environment, heat shock protein 70 (HSP70) has emerged as an endogenous cytoprotective factor that supports protein homeostasis, modulates apoptosis, and can attenuate inflammatory and oxidative tissue injury in experimental models of sepsis and lung damage [20,21,22]. When protective responses are inadequate, vascular leakage, alveolar flooding, and progressive impairment of pulmonary gas exchange may follow, which are hallmarks of sepsis-associated ALI.

Fenofibrate is a peroxisome proliferator-activated receptor-α (PPARα) agonist that is widely used to treat dyslipidemia and increasingly studied for anti-inflammatory and antioxidant properties [19]. In lung-relevant experimental settings, PPARα activation with fibrate ligands attenuates endotoxin-induced acute lung injury and reduces vascular leakage [23,24]. Fenofibrate suppresses LPS-induced airway inflammation in mice by reducing inflammatory cell influx and lowering cytokine and chemokine levels within lung compartments [25]. In an indirect ALI model triggered by intestinal ischemia–reperfusion, fenofibrate improved lung histopathology, inhibited NF-κB p65 activation, and reduced iNOS/NO signaling, supporting combined anti-inflammatory and redox-modulating actions [26]. Fenofibrate has also been reported to attenuate bleomycin-induced lung injury and fibrosis, with decreases in lung water content and TGF-β1 levels [27]. Mechanistically, fenofibrate has been linked to activation of Keap1-Nrf2 signaling and induction of antioxidant programs [28], and Nrf2 is broadly implicated in respiratory disease models, including ALI/ARDS [29]. However, fenofibrate’s effects in polymicrobial sepsis-associated acute lung injury remain insufficiently characterized.

To investigate fenofibrate in sepsis-associated ALI, we employed the FIP model of polymicrobial sepsis, which reflects colon-origin abdominal sepsis encountered in general surgery, such as colonic perforation or anastomotic leakage, through intraperitoneal administration of a standardized fecal slurry. Compared with cecal ligation and puncture, FIP offers improved reproducibility and more controlled disease severity while reliably inducing systemic inflammation and secondary lung injury, making it suitable for studying surgery-related sepsis and associated ALI [14]. Using a 24 h post-insult window, we evaluated fenofibrate’s effects with complementary readouts (biochemical markers, histopathology, and computed tomography), with particular emphasis on Nrf2 and HSP70 as candidate cytoprotective pathways.

## 2. Results

### 2.1. Histopathological Evaluation of Lung Injury

**Figure 1:**
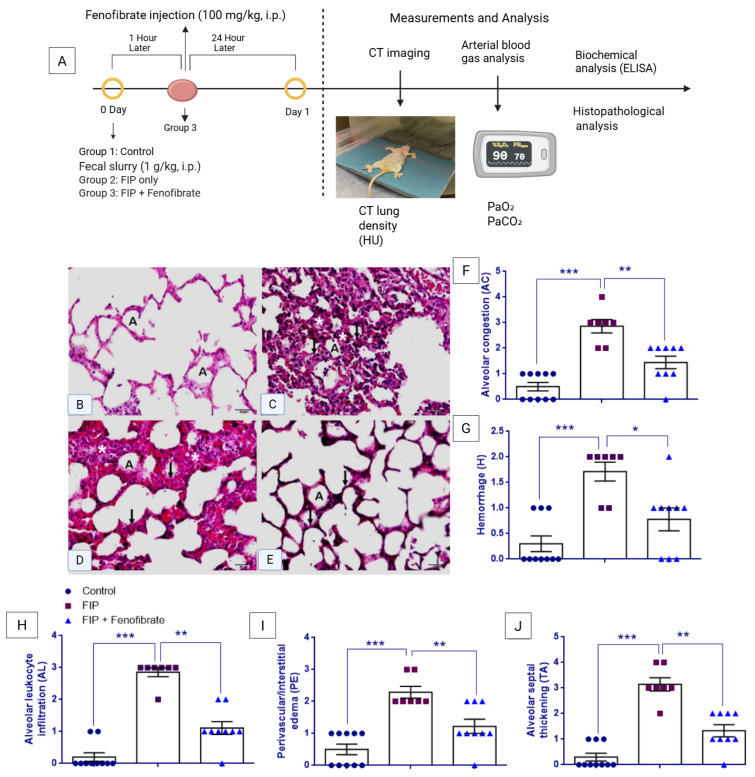
(**A**) Experimental design and lung injury assessment in the feces-induced peritonitis (FIP) model. Male Wistar rats (*n* = 30) were randomly assigned to Control, FIP, and FIP + Fenofibrate groups. Sepsis was induced by intraperitoneal injection of fecal slurry (1 g/kg). Fenofibrate (100 mg/kg, i.p.) was administered 1 h after FIP induction. All evaluations were performed at 24 h, including CT imaging, arterial blood gas analysis, biochemical assays, and histopathological examination. (**B**–**E**) Representative H&E-stained lung sections (×40). The control group shows preserved alveolar architecture, whereas the FIP group exhibits inflammatory infiltration and alveolar septal thickening. Fenofibrate treatment partially attenuated these histopathological alterations. Scale bar = 20 µm. **Symbols:** A: Alveolus; *: Inflammatory cell infiltration; arrows: Alveolar septal thickening. (**F**–**J**) Quantitative lung injury scores: alveolar congestion (AC), hemorrhage (H), alveolar leukocyte infiltration (AL), perivascular/interstitial edema (PE), and alveolar septal thickening (TA). Data are presented as mean ± SEM. Non-parametric analyses were performed using Kruskal–Wallis followed by Mann–Whitney U tests. * *p* < 0.05, ** *p* < 0.01, *** *p* < 0.001.

As shown in Figure 1F–J, histopathological scoring revealed significant group differences for alveolar congestion (AC), hemorrhage (H), alveolar leukocyte infiltration (AL), perivascular/interstitial edema (PE), and alveolar septal thickening (TA) (Kruskal–Wallis test: χ^2^ = 17.595, *p* < 0.001; χ^2^ = 13.054, *p* = 0.001; χ^2^ = 20.059, *p* < 0.001; χ^2^ = 16.239, *p* < 0.001; and χ^2^ = 19.080, *p* < 0.001, respectively). All histopathological scores were significantly higher in the FIP group than in the control group (Mann–Whitney U test: *p* < 0.001 for all). Following fenofibrate treatment, AC (*p* = 0.003), H (*p* = 0.012), AL (*p* = 0.001), PE (*p* = 0.006), and TA (*p* = 0.001) scores were significantly reduced compared with the FIP group; however, AC (*p* = 0.009), AL (*p* = 0.003), PE (*p* = 0.024), and TA (*p* = 0.004) scores remained elevated relative to controls, whereas hemorrhage scores did not differ significantly from control values (*p* = 0.096).

### 2.2. CT Hounsfield Unit (HU) Analysis

**Figure 2:**
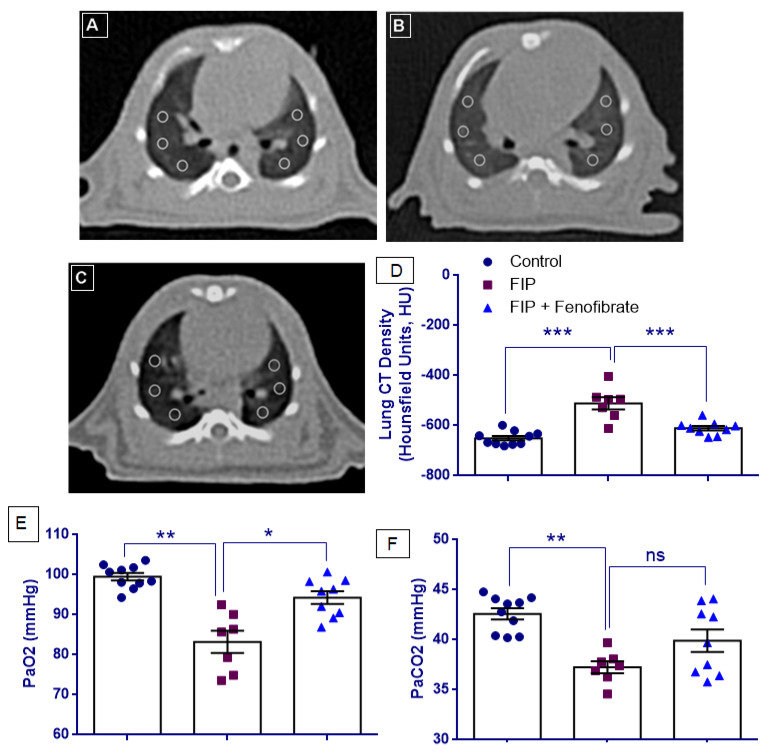
Computed tomography (CT) assessment of lung injury and arterial blood gas analysis in the feces-induced peritonitis (FIP) model. (**A**–**C**) Representative axial CT images of the lungs obtained at the level of the heart from the Control, FIP, and FIP + Fenofibrate groups, respectively. Six regions of interest (ROIs) of identical size were placed at standardized anatomical locations in both lungs for quantitative density analysis. The FIP group shows increased lung density compared with the Control group, whereas fenofibrate treatment partially restored lung aeration. (**D**) Quantitative analysis of lung density expressed as CT Hounsfield units (HU). Lower (more negative) HU values indicate greater lung aeration, whereas higher HU values indicate increased lung consolidation. (**E**,**F**) Arterial blood gas parameters, including partial pressure of oxygen (PaO_2_) and partial pressure of carbon dioxide (PaCO_2_) in Control, FIP, and FIP + Fenofibrate groups. Data are presented as mean ± SEM (Control, *n* = 10; FIP, *n* = 7; FIP + Fenofibrate, *n* = 9). Statistical analyses were performed using one-way ANOVA followed by Tukey’s HSD or Tamhane’s T2 post hoc tests, as appropriate. * *p* < 0.05, ** *p* < 0.01, and *** *p* < 0.001 indicate significant differences between the indicated groups.

As illustrated in Figure 2A–C, axial CT images demonstrate increased lung density in the FIP group compared with controls, whereas fenofibrate treatment is associated with a visible reduction in lung density, indicating partial attenuation of lung involvement.

CT Hounsfield unit (HU) values showed normal distribution across all experimental groups (*p* > 0.05). Levene’s test confirmed homogeneity of variances (F = 2.890, *p* = 0.076); therefore, one-way ANOVA followed by Tukey’s HSD post hoc test was applied. One-way ANOVA revealed a statistically significant group effect for CT HU values (F(2,23) = 24.564, *p* < 0.001). Post hoc analysis demonstrated that CT HU values were significantly increased in the FIP group compared with the control group (*p* < 0.001). Treatment with fenofibrate significantly reduced CT HU values compared with the FIP group (*p* < 0.001), whereas the difference between the FIP + Fenofibrate and control groups did not reach statistical significance (*p* = 0.105) (Figure 2D).

### 2.3. Analysis of Arterial Blood Gas Parameters

Arterial blood gas analysis revealed significant differences among groups for both PaO_2_ and PaCO_2_. Levene’s test indicated heterogeneity of variances for PaO_2_ (F = 6.642, *p* = 0.005), whereas homogeneity of variances was observed for PaCO_2_ (F(2,23) = 2.719, *p* = 0.087). Accordingly, one-way ANOVA followed by Tamhane’s T2 post hoc test was applied for PaO_2_, and one-way ANOVA followed by Tukey’s HSD post hoc test was used for PaCO_2_. One-way ANOVA demonstrated a significant group effect for PaO_2_ (F(2,23) = 21.671, *p* < 0.001) and for PaCO_2_ (F(2,23) = 7.377, *p* = 0.003) (Figure 2E).

PaO_2_ and PaCO_2_ levels were both significantly decreased in the FIP group compared with controls (*p* = 0.002 for both). Fenofibrate treatment significantly improved PaO_2_ levels compared with the FIP group (*p* = 0.019). In contrast, although PaCO_2_ levels showed a partial increase in the FIP + fenofibrate group compared with the FIP group, this change did not reach statistical significance (*p* = 0.177). Despite fenofibrate treatment, PaO_2_ levels remained significantly lower than those in the control group (*p* = 0.042), whereas PaCO_2_ levels in the FIP + fenofibrate group were not significantly different from control values (*p* = 0.119) (Figure 2F).

### 2.4. Plasma IL-6, IL-1β, and TNF-α Levels

**Figure 3:**
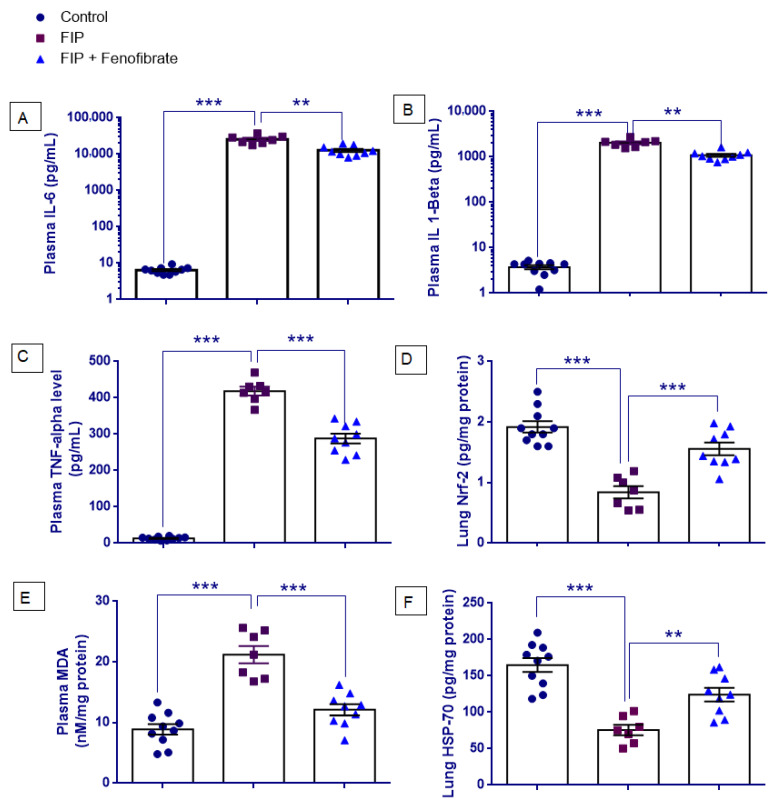
Effects of fenofibrate on inflammatory and oxidative stress-related markers in the feces-induced peritonitis (FIP) model. (**A**–**C**) Plasma inflammatory cytokine levels, including IL-6, IL-1β, and TNF-α, in Control, FIP, and FIP + Fenofibrate groups. IL-6 and IL-1β values are presented on a log_10_ scale due to their wide dynamic range. (**D**–**F**) Oxidative stress-related markers, including Nrf2, malondialdehyde (MDA), and HSP70 levels. Data are presented as mean ± SEM (Control, *n* = 10; FIP, *n* = 7; FIP + Fenofibrate, *n* = 9). Statistical analyses were performed using one-way ANOVA followed by appropriate post hoc tests. ** *p* < 0.01, *** *p* < 0.001 indicate significant differences between the indicated groups.

Figure 3A–C presents plasma IL-6, IL-1β, and TNF-α levels. All variables met the normality assumption (*p* > 0.05). However, Levene’s test indicated heterogeneity of variances for IL-6 (F(2,23) = 14.387, *p* < 0.001), IL-1β (F(2,23) = 8.998, *p* = 0.001), and TNF-α (F(2,23) = 11.295, *p* < 0.001). Therefore, one-way ANOVA followed by Tamhane’s T2 post hoc test was performed. One-way ANOVA demonstrated a significant overall group effect for: IL-6: F(2,23) = 78.197, *p* < 0.001, IL-1β: F(2,23) = 134.819, *p* < 0.001, TNF-α: F(2,23) = 455.999, *p* < 0.001.

Post hoc analyses showed that plasma IL-6, IL-1β, and TNF-α levels were significantly increased in the FIP group compared with the control group (*p* < 0.001 for all). Fenofibrate treatment significantly reduced IL-6, IL-1β, and TNF-α levels compared with the FIP group (*p* = 0.005, *p* = 0.001, and *p* < 0.001, respectively). Nevertheless, IL-6, IL-1β, and TNF-α levels in the FIP + Fenofibrate group remained significantly higher than those observed in the control group (*p* < 0.001 for all).

### 2.5. Plasma MDA and Lung Nrf2 Levels

As shown in Figure 3D,E, Shapiro–Wilk analysis demonstrated that Nrf2 and MDA levels were normally distributed across all experimental groups (*p* > 0.05). Levene’s test confirmed homogeneity of variances for both Nrf2 (F = 0.253, *p* = 0.779) and MDA (F = 1.285, *p* = 0.296); therefore, one-way ANOVA followed by Tukey’s HSD post hoc test was applied. One-way ANOVA revealed a statistically significant group effect for Nrf2 (F(2,23) = 27.886, *p* < 0.001) and MDA (F(2,23) = 34.473, *p* < 0.001). Post hoc analysis showed that Nrf2 levels were significantly decreased in the FIP group compared with the control group (*p* < 0.001), while fenofibrate treatment significantly increased Nrf2 levels compared with the FIP group (*p* < 0.001). However, Nrf2 levels in the FIP + Fenofibrate group remained significantly lower than control values (*p* = 0.034). In contrast, MDA levels were significantly increased in the FIP group compared with the control group (*p* < 0.001), and fenofibrate treatment significantly reduced MDA levels in the FIP + Fenofibrate group compared with the FIP group (*p* < 0.001), whereas the difference between the FIP + Fenofibrate and control groups did not reach statistical significance (*p* = 0.079).

### 2.6. Pulmonary HSP70 Levels

As shown in Figure 3F, HSP70 data met the assumptions of normality and homogeneity of variance (Shapiro–Wilk *p* > 0.05; Levene’s F = 1.433, *p* = 0.259); therefore, one-way ANOVA with Tukey’s HSD post hoc test was applied. One-way ANOVA revealed a statistically significant group effect for HSP70 levels (F(2,23) = 22.513, *p* < 0.001). Post hoc analysis demonstrated that HSP70 levels were significantly decreased in the FIP group compared with the control group (*p* < 0.001). Fenofibrate treatment significantly increased HSP70 levels compared with the FIP group (*p* = 0.005); however, levels remained significantly lower than those in the control group (*p* = 0.009).

## 3. Discussion

In the present study, fenofibrate provided significant protection against acute lung injury associated with feces-induced peritonitis, a clinically relevant model of polymicrobial abdominal sepsis. These findings indicate that fenofibrate improves both structural and functional lung parameters in experimental polymicrobial sepsis.

In this experimental model, untreated FIP rats exhibited severe histopathological lung injury, characterized by alveolar congestion and hemorrhage, prominent leukocyte infiltration, perivascular edema, and marked alveolar wall thickening with hyaline membrane formation, indicating extensive capillary leakage and diffuse alveolar damage. These findings align with previous reports showing that polymicrobial sepsis induces severe sepsis-associated acute lung injury [14,30,31,32]. Fenofibrate treatment markedly attenuated these pathological changes, significantly reducing congestion, inflammatory cell infiltration, edema, and alveolar wall thickening. This protective effect is consistent with prior ALI studies demonstrating that fenofibrate improves lung histology by reducing edema and cellular infiltration [33]. Importantly, these structural improvements were accompanied by functional benefits, as CT analysis showed restoration of lung aeration, reflected by more negative Hounsfield unit values. These findings suggest that fenofibrate preserves alveolar-capillary integrity and mitigates microvascular injury in sepsis.

Previous studies have shown that arterial blood gas parameters, particularly reductions in PaO_2_, reflect pulmonary dysfunction in sepsis-associated acute lung injury and ARDS [16,32,34]. Consistent with these reports, our FIP-induced sepsis model showed a marked decrease in PaO_2_, indicating impaired alveolar gas exchange. Fenofibrate treatment significantly improved arterial oxygenation and partially reversed sepsis-associated hypocapnia, suggesting normalization of ventilatory drive. These functional changes were corroborated by CT and histological findings, which showed attenuation of lung density changes, alveolar congestion, edema, and wall thickening.

Sepsis is characterized by an uncontrolled systemic inflammatory response driven by excessive cytokine release, including TNF-α, IL-1β, and IL-6, as consistently shown in both preclinical and clinical studies [14,35,36]. This cytokine surge disrupts the alveolo-capillary barrier by increasing microvascular permeability, ultimately leading to acute lung injury [16,34]. In our study, the FIP model reproduced these hallmarks of sepsis-induced ALI, including alveolar injury, edema, and neutrophilic accumulation. Feno-fibrate significantly lowered IL-6, IL-1β, and TNF-α levels in septic rats. Similarly, CLP-based studies demonstrated fenofibrate’s capacity to suppress NF-κB-mediated cytokine expression [14,37]. Beyond the lung, fenofibrate also showed protective effects in other organs: Zeng et al. demonstrated enhanced mitochondrial function and reduced renal inflammation in septic AKI [38], and Lv et al. highlighted attenuation of cardiac oxidative stress and cytokine release [39]. These findings support fenofibrate’s multi-organ protective role in sepsis and align mechanistically with our pulmonary observations.

Accumulating evidence indicates that oxidative stress is a central mediator of sepsis-induced ALI, driven by excessive ROS production from infiltrating neutrophils and dysfunctional mitochondria [3,40,41]. In our FIP model, elevated malondialdehyde (MDA) levels indicated increased lipid peroxidation, while reduced nuclear factor erythroid 2-related factor 2 (Nrf2) levels reflected impaired antioxidant defenses [42,43]. Fenofibrate treatment significantly decreased MDA and partially restored Nrf2 levels, suggesting reactivation of endogenous cytoprotective mechanisms. These findings align with previous reports showing that fenofibrate enhances antioxidant defenses and reduces inflammation in various organ injury models [37,39,44,45]. Nrf2 is a key transcriptional regulator of antioxidant genes, and its activation enhances the expression of heme oxygenase-1 (HO-1) and glutathione-dependent enzymes, responses shown to mitigate oxidative damage in sepsis models [43,46,47]. Our data support the hypothesis that fenofibrate strengthens Nrf2-driven antioxidant signaling, thereby contributing substantially to its protective effects in septic lung injury.

A novel finding in our study was the marked suppression of heat shock protein 70 (HSP70) in the lungs of FIP-induced septic rats, indicating impaired cellular stress responses during polymicrobial sepsis. HSP70, a chaperone that stabilizes proteins and prevents apoptosis, declines under severe inflammatory conditions such as sepsis [48]. Notably, fenofibrate was associated with increased pulmonary HSP70 levels in our model. This is consistent with Colunga Biancatelli et al. [49], who showed that HSP70 enhancement improves tolerance to chemical lung injury. Supporting this, prior studies have demonstrated that HSP70 protects against sepsis-induced lung injury by suppressing NF-κB signaling, reducing cytokine release, and limiting oxidative stress [20,21,50,51]. Additionally, preserving or restoring HSP70 expression was shown to reduce vascular leakage, protect alveolar-capillary barrier integrity, and improve survival in rodent sepsis models [52,53].

Previous studies have indicated that cytoprotective pathways such as Nrf2 and HSP70 do not always increase during severe sepsis. Sepsis is characterized by a dysregulated host response in which hyperinflammation and immune suppression may coexist. Under intense inflammatory stress, these protective responses can become impaired or exhausted. Experimental studies have shown that pulmonary heat-shock responses may be diminished in CLP-induced sepsis models, where lung HSP70 expression fails to increase despite severe inflammatory injury [51]. Similarly, Nrf2 signaling may be functionally suppressed during endotoxin-driven inflammation due to impaired transcriptional activation and disrupted redox signaling [47,54]. Therefore, the reduced Nrf2 and HSP70 levels observed in our FIP group likely reflect a dysregulated cytoprotective response rather than an absence of oxidative stress, a notion supported by the marked increase in lipid peroxidation (MDA). In this context, fenofibrate may act not by excessively activating stress pathways, but by partially restoring suppressed cytoprotective defenses while simultaneously reducing oxidative injury.

Recent evidence indicates that Nrf2 may influence HSP70 expression through interconnected stress-response pathways [55,56,57,58]. In sepsis, impaired Nrf2 signaling can weaken cellular stress tolerance and antioxidant defenses. Consistent with this, our study showed reduced pulmonary Nrf2 and HSP70 levels in FIP-treated rats, suggesting suppression of cytoprotective responses during polymicrobial sepsis. Although fenofibrate has been reported to activate the PPARα/Nrf2 axis in various organs [37,38,39], direct evidence linking it to HSP70 induction is limited. Thus, the increased HSP70 levels observed after fenofibrate treatment in our model likely reflect indirect restoration of Nrf2-dependent cytoprotective signaling rather than direct pharmacological activation of HSP70.

The present study findings may be explained by the anti-inflammatory and antioxidant effects mediated through PPARα. Activation of PPARα has been shown to suppress NF-κB signaling and reduce pro-inflammatory cytokine production during inflammatory stress [59]. In addition, fenofibrate has been reported to enhance antioxidant enzyme activity and modulate Nrf2-related redox pathways in experimental models [19,28,29]. Consistent with these mechanisms, experimental studies demonstrate that fenofibrate attenuates oxidative stress and inflammatory lung injury through suppression of NF-κB signaling and reduction in oxidative damage [26]. These pathways suggest that fenofibrate may limit sepsis-induced lung injury by simultaneously reducing inflammatory signaling and restoring cytoprotective antioxidant defenses.

Fenofibrate was administered 1 h after FIP induction to model an early post-insult therapeutic intervention rather than prophylaxis. Polymicrobial peritonitis can trigger systemic inflammatory signaling within the first hour, including rapid increases in circulating TNF-α following fecal inoculum [60]. Sepsis guidelines similarly emphasize initiating therapy as soon as sepsis is recognized, ideally within the first hour [61]. Moreover, the FIP model has been shown to reproducibly induce systemic inflammation and acute lung injury within 24 h, reflecting early stages of sepsis-associated ALI in experimental settings. Because ARDS/ALI evolves over hours to days and most clinical cases are identified within 72 h of the inciting insult [11], early modulation of inflammatory pathways may attenuate subsequent lung injury endpoints measured at 24 h. Nevertheless, delayed-dosing studies are warranted to further clarify the therapeutic window of fenofibrate.

### Limitations

A limitation of this study is the absence of a fenofibrate-only control group without FIP induction, which would have helped distinguish the direct pharmacological effects of fenofibrate from its protective effects under septic conditions. Only male rats were included to minimize variability related to the estrous cycle and hormonal fluctuations. Therefore, potential sex-related differences in inflammatory and oxidative stress responses could not be evaluated, and future studies including both sexes are warranted.

## 4. Materials and Methods

### 4.1. Animals

This study was conducted using 30 adult male Wistar albino rats, weighing between 200 and 250 g. All experimental procedures complied with the National Institutes of Health Guide for the Care and Use of Laboratory Animals. Ethical approval was obtained from the Animal Ethics Committee of Science University (Approval No: 13211004).

The animals were supplied by the Experimental Animal Research Laboratory of Science University. Rats were housed in pairs in stainless-steel cages under controlled environmental conditions (22 ± 2 °C) with a 12 h light/dark cycle. Standard laboratory chow and water were provided ad libitum throughout the study.

### 4.2. Drugs and Chemicals

Anesthesia was induced by intraperitoneal administration of ketamine (Ketasol^®^, 75 mg/kg) and xylazine (Rompun^®^, 15 mg/kg), obtained from commercial suppliers (Richter Pharma AG, Wels, Austria; Bayer, Leverkusen, North Rhine-Westphalia, Germany). Fenofibrate (Sigma-Aldrich, St. Louis, MO, USA) was administered intraperitoneally at a dose of 100 mg/kg, consistent with doses used in rodent models of sepsis and ischemia–reperfusion injury [26,39]. Fenofibrate was first dissolved in DMSO and subsequently diluted with distilled water to obtain a final vehicle containing 10% DMSO. The final injection volume was 1 mL. To ensure consistency, rats in the control and FIP groups received the same intraperitoneal injection volume (1 mL) of vehicle (10% DMSO and 90% distilled water) as the fenofibrate-treated group.

### 4.3. Experimental Procedures

Rats were randomly allocated into three experimental groups. A feces-induced peritonitis (FIP) model was applied to induce experimental sepsis in 20 rats, while 10 rats served as normal controls and received no intervention.

The FIP model was established according to previously described protocols by Shrum et al. [62] and Tyml et al. [63]. Fresh feces were collected and homogenized in sterile saline to prepare the fecal suspension. This suspension was administered intraperitoneally at a dose of 1 g/kg body weight.

The experimental groups were established as described below (Figure 1A).

Study groups:

Group 1: Normal Control (*n* = 10)—Rats received no surgical or pharmacological intervention and were fed orally under standard conditions.

Group 2: FIP Group (*n* = 10)—Rats underwent the FIP procedure and received no additional treatment.

Group 3: FIP + Fenofibrate Group (*n* = 10)—Rats underwent the FIP procedure and were treated with fenofibrate (100 mg/kg/day), administered intraperitoneally (i.p).

All treatments were initiated one hour after the FIP procedure. The experimental period was limited to 24 h.

During the first 24 h following FIP induction, four rats died and were excluded from further analysis (three from the FIP group and one from the fenofibrate-treated group).

At the end of the experimental period, animals were anesthetized with ketamine (75 mg/kg) and xylazine (15 mg/kg). Under anesthesia, thoracic CT imaging was first performed, followed by arterial blood gas analysis. Blood samples were then collected via cardiac puncture for biochemical analyses. Subsequently, transcardiac perfusion was performed, and lung tissues were excised for histopathological evaluation.

### 4.4. Biochemical Analysis

#### 4.4.1. Determination of TNF-α, IL-6, and IL-1β Levels in Plasma

Blood samples were collected by cardiac puncture and centrifuged at 3000× *g* for 10 min to obtain plasma. Plasma samples were immediately snap-frozen in liquid nitrogen and stored at −80 °C until analysis. Plasma levels of tumor necrosis factor-α (TNF-α), interleukin-6 (IL-6), and interleukin-1β (IL-1β) were measured using commercially available rat-specific enzyme-linked immunosorbent assay (ELISA) kits (Abcam, Cambridge, UK; TNF-α: ab236712; IL-6: ab100772; IL-1β: ab100767), according to the manufacturers’ instructions. Plasma samples were diluted 1:2 prior to analysis, and all measurements were performed in duplicate.

#### 4.4.2. Lung Biochemical Analysis of HSP70 and Nrf2

Following euthanasia, lung tissues were rapidly excised and stored at −80 °C until analysis. For biochemical evaluation, lung samples were homogenized in ice-cold phosphate-buffered saline (PBS, pH 7.4) using a glass homogenizer at a ratio of 1:5 (*w*/*v*). Homogenates were centrifuged at 5000× *g* for 15 min at 4 °C, and the supernatants were collected for analysis.

Total protein concentrations were determined using the Bradford method with bovine serum albumin as the standard. Lung tissue HSP70 and Nrf2 levels were quantified from tissue supernatants using commercially available rat-specific ELISA kits (MyBioSource, San Diego, CA, USA; HSP70: MBS035085; Nrf2: MBS752046), in accordance with the manufacturers’ instructions. Analyte concentrations were calculated from standard curves, expressed as pg/mL, and normalized to total protein content (pg/mg protein).

#### 4.4.3. Measurement of Lipid Peroxidation

Plasma lipid peroxidation was assessed by determining malondialdehyde (MDA) levels using the thiobarbituric acid reactive substances (TBARS) method. Plasma samples were reacted with trichloroacetic acid and TBARS reagent, followed by thorough mixing. The mixtures were heated at 100 °C for 60 min to allow color development.

After incubation, samples were cooled on ice and centrifuged at 3000 rpm for 20 min. The absorbance of the clear supernatant was then measured spectrophotometrically at 535 nm, and MDA levels were calculated accordingly [64].

### 4.5. Histopathological Examination of Lung

Animals were deeply anesthetized prior to tissue harvest. Transcardiac perfusion fixation was performed as a terminal procedure [65] by flushing the vasculature with phosphate-buffered saline (PBS), followed by perfusion with 4% paraformaldehyde (PFA) in 0.1 M phosphate buffer. After perfusion, lungs were excised and immersion-fixed in 10% neutral-buffered formalin (NBF). Because 10% NBF and 4% PFA are both formaldehyde-based fixatives with comparable effective formaldehyde concentrations, their combined use for perfusion followed by immersion fixation is methodologically acceptable when clearly reported [66].

Fixed lungs were processed for paraffin histology using graded ethanol dehydration, xylene clearing, and paraffin infiltration/embedding according to standard tissue-processing workflows (Abcam, Cambridge, UK, 2017). Briefly, tissues were dehydrated through graded ethanol solutions (70%, 80%, 95/96%, and 100%), cleared in xylene, and infiltrated with paraffin wax at 56–58 °C before embedding. Sections were cut at 5 µm thickness using a rotary microtome and mounted on glass slides.

#### 4.5.1. Hematoxylin and Eosin Staining

Paraffin sections were deparaffinized in xylene and rehydrated through descending ethanol concentrations to distilled water. Sections were stained with Mayer’s hematoxylin (Sigma-Aldrich, USA), followed by bluing under running tap water and counterstaining with eosin Y. Slides were then dehydrated through ascending ethanol concentrations, cleared in xylene, and coverslipped using a resinous mounting medium, consistent with H&E staining procedures.

#### 4.5.2. Microscopy and Image Acquisition

Histological images were obtained using an Olympus BX51 light microscope (Olympus Corporation, Tokyo, Japan) coupled to an Olympus C-5050 digital camera (Olympus Corporation, Tokyo, Japan). Representative images shown in Figure 1B–E were captured using a 40× objective lens (total magnification 400× with a 10× ocular). Scale bars were generated after microscope-to-camera calibration using a stage micrometer.

#### 4.5.3. Histopathological Scoring

Histopathological lung injury was assessed on hematoxylin–eosin (H&E)-stained sections using a semi-quantitative scoring system commonly applied in experimental ALI studies [12,13,67]. The following parameters were evaluated: alveolar congestion (AC), hemorrhage (H), leukocyte infiltration or aggregation in air spaces or vessel walls (AL), perivascular/interstitial edema (PE), and alveolar septal thickening and/or hyaline membrane formation (TA). Each parameter was graded using a 0–4 scoring scale based on the proportion of tissue involvement: 0 = no pathological change; 1 ≤ 25%; 2 = 26–50%; 3 = 51–75%; and 4 > 75%. Histopathological evaluation was performed independently by a blinded histopathologist.

### 4.6. CT Examination of the Lung

All CT scans were performed using a 16-slice multidetector CT scanner (Somatom Go Now, Siemens Healthcare, Erlangen, Germany). Imaging was conducted in the supine position without the use of contrast material.

To minimize motion artifacts, animals were secured to the scanning table using appropriate restraint materials. Scanning parameters included 120 kV, automatic exposure-controlled mAs, and a slice thickness of 1 mm. The scan range extended from the C3 vertebra to the diaphragm, covering both the apical and basal lung regions.

Following image acquisition, axial images were reconstructed as non-overlapping 1 mm slices using a 512 × 512 matrix and a sharp reconstruction algorithm (Kernel Br64; (Siemens Healthineers, Erlangen, Germany).

All images were independently evaluated by two experienced radiologists (B.Ö. and İ.H.S.) who were blinded to the experimental groups and laboratory data. Quantitative analysis was performed by placing six regions of interest (ROIs) of identical size (2153 mm^2^) on axial lung images using the parenchymal window. ROIs were distributed as two in the upper, two in the middle, and two in the lower lung zones. Care was taken to avoid large vessels, airways, and bony structures during ROI placement.

### 4.7. Arterial Blood Gas Analysis

Arterial blood gas analysis was performed 24 h after the experimental procedure. For this purpose, arterial blood samples (approximately 0.2 mL) were collected from the carotid artery of rats in each group under appropriate anesthesia. Special care was taken to avoid air contamination during sampling.

Blood samples were immediately analyzed using an automated blood gas analyzer. Partial arterial oxygen pressure (PaO_2_) and partial arterial carbon dioxide pressure (PaCO_2_) values were recorded for each animal and used for subsequent comparisons between groups.

### 4.8. Statistical Analysis

All statistical evaluations were carried out using SPSS software (version 19; IBM Corp., Armonk, NY, USA). The normality of data distribution was examined using the Shapiro–Wilk test, while variance homogeneity was assessed by Levene’s test.

Normally distributed variables are reported as mean ± standard error of the mean (SEM) and were compared among groups using one-way analysis of variance (ANOVA). When a significant overall effect was detected, Tukey’s post hoc test was applied for multiple group comparisons.

Data that did not meet parametric assumptions, including histopathological lung injury scores, are presented as median [interquartile range, IQR]. These variables were analyzed using the Kruskal–Wallis test, followed by pairwise comparisons with the Mann–Whitney U test adjusted by Bonferroni correction. Statistical significance was defined as a *p*-value less than 0.05.

## 5. Conclusions

The present study demonstrates that fenofibrate effectively attenuates feces-induced peritonitis-associated acute lung injury by improving pulmonary gas exchange, restoring lung aeration, and preserving histopathological integrity. These protective effects are accompanied by suppression of inflammatory and oxidative stress responses, indicating a coordinated structural and functional lung-protective action in polymicrobial sepsis representative of colon-origin abdominal infection. These findings may be explained by the anti-inflammatory and antioxidant effects mediated through PPARα activation.

## Data Availability

The data that support the findings of this study are not publicly available due to ethical reasons; however, they are available from the corresponding author upon request.
